# Complete mitochondrial genome of *Sesia siningensis* (Lepidoptera: Sesiidae)

**DOI:** 10.1080/23802359.2019.1703583

**Published:** 2019-12-18

**Authors:** Jingyan Yan, Daoxin Liu, Pengfei Song, Shou Feng, Wangyan Li, Ying Li

**Affiliations:** a State Key Laboratory of Plateau Ecology and Agriculture, Qinghai University, Xining, China;; b College of Agriculture and Animal Husbandry, Qinghai University, Xining, China;; c Qinghai Provincial Key Laboratory of Animal Ecological Genomics, Xining, China

**Keywords:** *Sesia siningensis*, Sesiidae, mitogenome, clearwing moth

## Abstract

*Sesia siningensis* is an important trunk borer of poplar and is widely distributed in China. Here, the complete mitochondrial genome of *S. siningensis* was sequenced. The circle genome of the clearwing moth is 15,454 bp in length. There are 38 sequence elements including 13 protein coding genes, 22 tRNA genes, 2 rRNA genes, and a control region. The order of most elements was consistent with that of *Chilo suppressalis*, with the exception of one tRNA gene. As the first reported mitochondrial genome in the Sesiidae family, it will provide useful information to the development and application of better markers and primers in the molecular taxonomy of this family.

The poplar-trunk clearwing moth, *Sesia siningensis*, is a destructive trunk borer of poplar, willow and even pagoda tree and is widely distributed in China (Ma et al. 2003). Due to its great damage in urban landscapes, timber stands and artificial shelter forests, and the economic loss, *S. siningensis* is listed as an object of China forestry plant quarantine (Li 2009). Just as the poplar-trunk clearwing moth, many species in the Sesiidae family are important forest pests and can result in serious economic loss by larval boring in stems and roots of herbaceous and woody plants (Nielson 1978; McKern and Szalanski 2007). To reach a deeper knowledge about this family and to benefit the species-specific monitoring and control programs, many studies have been focused on the molecular classification and identification of clearwing moths (McKern et al. 2008; Hansen et al. 2012; Taft and Cognato 2017). Here, we sequenced the complete mitochondrial genome of *S. siningensis* (GenBank accession number: MN708363), which is the first reported mitochondrial genome in the Sesiidae family and will provide useful information to the development and application of better markers and primers in the molecular taxonomy of this family.

In this study, *S. siningensis* adults were collected from Southern Mountains in Xining, Qinghai Province, China (36°37′36″ N, 101°44′58″ E) in August 2019 and stored in the Insect Collection of the Entomology Lab, College of Agriculture and Animal Husbandry, Qinghai University, Xining, China (accession number: YJY-2019-001). Genomic DNA was extracted from a single sample and Genomic sequencing was performed on the Illumina HiSeq Platform (Illumina, San Diego, CA) with a read length of 150 bp. Approximately 50 GB of clean data were finally yielded. The NOVOPlasty v2.7.2 program (Dierckxsens et al. 2017) was employed to assemble the mitogenome. The assembled sequence was then annotated using the web server MITOS (Bernt et al. 2013).

A circularized DNA assembly 15,454 bp in length was harvested and deposited in the GenBank with accession number of MN708363. Like many other moth mitogenomes, 38 sequence elements are recognized in this mitogenome: 13 protein-coding genes (PCGs), 22 transfer RNA (tRNA) genes, two ribosomal RNA (rRNA) genes, and an AT rich control region(D-loop). Of all 38 sequence elements, 4 PCGs, 8 tRNA genes and 2 rRNA genes are located on the light strand, while others are on the heavy strand.

Based on mitochondrial genome sequences assembled here or downloaded from GenBank, phylogenetic relationships of *S. siningensis* with 10 other species, totally representing 9 families in Ditrysia, the Noctuidae, Erebidae, Geometridae, Sphingidae, Tortricidae, Gelechiidae, Crambidae, Oecophoridae and Sesiidae, were resolved by means of Neighbor-joining (Figure 1). Alignment was conducted using MAFFT (Katoh and Standley 2013). The Neighbor-joining tree was built using MEGA7 (Kumar et al. 2016) with bootstrap set to 1000. The phylogenetic tree showed that *S. siningensis*, *Lobesia botrana* and *Epiphyas postvittana* formed a monophyletic clade with 100% bootstrapping rates, indicating a closer relationship between the Sesiidae and Tortricidae.

**Figure 1.**
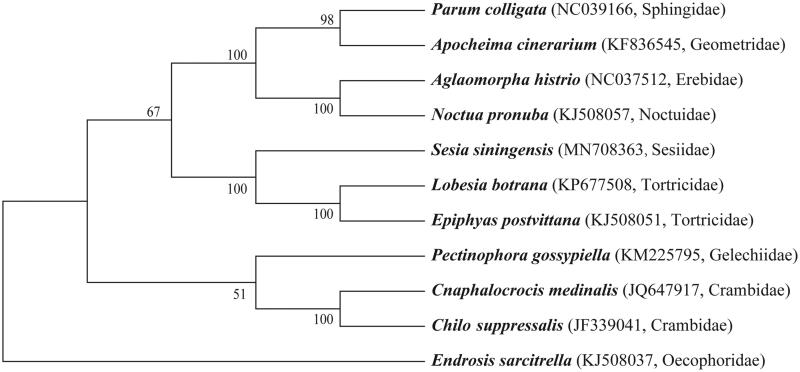
The Neighbor-joining tree based on 11 mitochondrial genome sequences.
